# Spatially resolved transcriptomics provide a new method for cancer research

**DOI:** 10.1186/s13046-022-02385-3

**Published:** 2022-05-19

**Authors:** Bowen Zheng, Lin Fang

**Affiliations:** Department of Breast and Thyroid Surgery, Shanghai Tenth People’s Hospital, School of Medicine, Tongji University, Shanghai, 200072 People’s Republic of China

**Keywords:** Spatially resolved transcriptomics, Cancer, Research Progress

## Abstract

A major feature of cancer is the heterogeneity, both intratumoral and intertumoral. Traditional single-cell techniques have given us a comprehensive understanding of the biological characteristics of individual tumor cells, but the lack of spatial context of the transcriptome has limited the study of cell-to-cell interaction patterns and hindered further exploration of tumor heterogeneity. In recent years, the advent of spatially resolved transcriptomics (SRT) technology has made possible the multidimensional analysis of the tumor microenvironment in the context of intact tissues. Different SRT methods are applicable to different working ranges due to different working principles. In this paper, we review the advantages and disadvantages of various current SRT methods and the overall idea of applying these techniques to oncology studies, hoping to help researchers find breakthroughs. Finally, we discussed the future direction of SRT technology, and deeper investigation into the complex mechanisms of tumor development from different perspectives through multi-omics fusion, paving the way for precisely targeted tumor therapy.

## Background

Despite many types of research done on cancer, it is still one of the diseases with a high mortality rate and seriously affects the quality of human life. In recent years, with the maturation and application of single-cell RNA sequencing (scRNA-seq) technology, significant breakthroughs have been made in the research of cancer signaling pathways, cytodynamics, and tumor microenvironment in various types of tumors [1, 2]. Especially in hematological malignancies, the application of this technique has made great contributions to the staging and treatment of leukemia [3]. However, it is far from enough to study only the differences in gene replication, transcription, and translation between cancer tissues and normal tissues. The heterogeneity of tumors is also manifested in the interaction of heterotypic cells and the regional enrichment of immune cells, which results in phenotypic differences in spatial dimensions [4, 5]. But for solid tumors, The homogenization of tissue before sequencing destroys the original spatial information [6]. To solve this issue, a method for nucleic acid analysis after partial lysis of adherent cells was presented [7]. Although the spatial specificity of tumor tissue cannot be accurately restored, it laid a foundation for the emergence of spatially resolved transcriptomics (SRT).

SRT technique was proposed based on past research and ongoing developments in computer approaches for sequencing findings. SRT combines RNA sequencing results with spatial backdrop information to help researchers better understand the roles of distinct cells and how they interact, opening up new avenues for biological research. SRT technologies have been employed in numerous biological investigations in recent years, with a great number of them being published, the majority of the study focusing on embryonic development [8, 9] and neuroanatomy [10–12], while there are still few cancer-related studies.

In this review, to help researchers choose the right technique, we discuss the different techniques included in SRT at this stage, as well as the basic working principles and the existence of benefits and drawbacks of these techniques, including differences in spatial resolution, sample handling, complexity, and throughput. A slew of hardware and software advances have aided in the growth of SRT analysis reports’ reliability. In addition, a plethora of web-based databases have been created for usage, substantially alleviating the cost concerns of those researchers. We also talk about how SRT has been used in cancer research so far, including xenomorphic cell interactions in tumor tissues, tumor immune microenvironment, tumor ecology, and so on. Finally, we considered potential avenues for future uses of SRT technology in the field of tumor research, including integrating multi-omics data to gain a greater understanding of tumor cellular modes of action, this will lead to breakthroughs in the discovery of more targeted treatments for tumors.

## Basic methods of spatially resolved transcriptomics

Different methods are classified into two groups based on how they encode spatial backdrop information: One is an imaging-based method, the transcriptome can be read in situ by special microscopes or in combination with fluorescence in situ hybridization (FISH) techniques [13], and the other is in situ barcoding-based methods, Spatial background information is saved into barcodes before non-in situ sequencing and combined with transcriptome information [14].

### Imaging-based spatially resolved transcriptomics

Imaging-based SRT enables high spatial resolution transcriptomic analysis. Behind the high resolution is the high demand for optical imaging capability and data processing ability. Generally, imaging-based methods are divided into two types: multiplexed fluorescence in situ hybridization (FISH) or based on in situ sequencing (ISS). The two methods are similar in some respects.

#### Multiplexed FISH

**Fig. 1:**
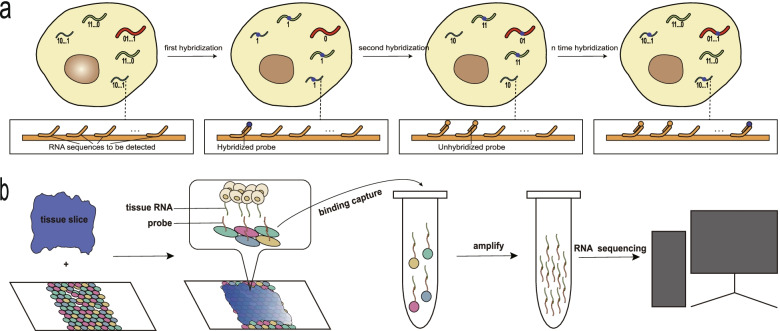
**a** A Multiplexed FISH strategy, achieves spatial information retention of the cell transcriptome through multiple rounds of FISH hybridization and binary computation. **b** In SPBC, tissues are placed on slides lined with beads containing gene probes containing positional information that can bind to tissue RNA. The tissue RNA is permeabilized onto the slide by the action of enzymes, and the tissue RNA that has bound to the probe is subsequently reverse transcribed into cDNA and sequenced

Traditional FISH methods are greatly limited in large-scale RNA detection due to limited color channels. Cai and his colleagues attempted to use enzymes to separate the already bound FISH probes and RNA to duplicate the utilization of color channels [15]. This method increased the number of detectable RNAs, although the effect is limited, it provides a new way of thinking. Excitingly, Zhuang and his team proposed a new method of multiplexed error-robust FISH (MERFISH) [16]. RNA is assigned a value of “0” or “1” based on whether it is found in one round of detection, and the number of detectable RNAs is exponentially increased by several rounds of hybridization and binary computations in the MERFISH method. (Fig. 1a) At the same time, it also leads to the infinite magnification of small errors in each round of testing. MERFISH employs coding systems that enable the identification and repair of errors [16]. In 2019, MERFISH was successfully used for large-scale RNA imaging with about 80% detection efficiency and about 4% false positives involving 23 cycles of hybridization and three-color imaging [17]. In addition, expansion microscopy helps reduce molecular crowding in MERFISH imaging applications [18, 19], and hydrogel tissue treatment reduces interference from lipids and proteins that interfere with probe-specific binding [20]. Based on MERFISH, improved seqFISH [21], ABER-FISH [22] and osmFISH [23] have been proposed to solve the issue of dense RNA molecular imaging. Researchers have used these techniques to generate medium-scale molecular resolution maps of RNA. Also, the mass production of recyclable probes reduces the cost of detection [24]. However, due to the complexity of dealing with tumor tissues and other technical reasons, none of them are widely used in cancer research in a commercialized form. In the future, Multiplexed FISH methods will likely be further applied to the quantitative analysis of low expression RNA due to their high detection efficiency.

#### In situ sequencing (ISS)

In situ sequencing can be done in two ways: targeted or untargeted. In targeted in situ sequencing, a particular nucleotide is utilized as a barcode to attach to a target gene, and the target sequence is executed rolling circle amplification (RCA) to acquire enough fluorescence intensity that can be recognized precisely by the device [25]. The STARmap method enables the direct binding of probes to target RNA. Simultaneously, it used hydrogel tissue processing technologies with signal amplification techniques to overcome the problem of low detection efficiency in complex tissues [12]. In untargeted in situ sequencing, the fluorescent in situ RNA sequencing (FISSEQ) method is representative, RNA is indiscriminately converted to cDNA, amplified, and sequenced. Amplification primers help limit the amount of RNA detected [26]. The non-targeted feature allows the FISSEQ method to achieve full gene coverage, but also creates inefficient detection. Technology firms are already mass-producing FISSEQ reagents and apparatus [27], which might be the first to be employed on a wide scale in tumor spatial transcriptomics investigations.

In addition, many improved in situ sequencing methods have been proposed by researchers. By combining in situ sequencing with non-in situ sequencing, the reduced accuracy of the results caused by sequencing large molecular weight RNAs has been settled [28]. or targeted sequencing, Zhuang mentioned a strategy in his comment [13], using the absence of signal to adjust the molecular density. This strategy combined with the use of expansion microscopy is expected to achieve efficient detection of large-scale RNA. For untargeted sequencing, we consider that we can keep some “invalid” RNA molecules from being detected by increasing the specificity of amplification primers and reducing molecular crowding.

### In situ barcoding-based spatially resolved transcriptomics

Several in situ DNA barcoding-based methods are being developed in parallel. All of these methods use DNA barcoding to gather spatial background information on genes, which is then matched with gene expression data using advanced algorithms to acquire tissue expression profiles at complete cellular resolution. The main advantage of these methods is complete capture of the entire transcriptome, although large-scale data processing is relatively the imaging-based methods more demanding algorithm.

In the fundamental methods termed solid phase-based capture (SPBC), spatial barcodes and RNA probes are printed and gridded on glass slides, then tissue sections are placed on a glass slide, RNA is released and hybridized to the probe by enzyme penetration. Subsequently, the new sequence formed by the RNA and the probe was reverse transcribed to cDNA and sequenced. Finally, spatial barcodes are mixed with the original tissue images to match [29, 30]. (Fig. 1b) 10X Genomics had already taken this technology from laboratory expertise to commercialization and improved the resolution of imaging, their new method was termed 10X Visium. The diameter of each well on the glass slide was reduced to 55 μm. They also increased the capture density to 5000 spots per slide by printing hexagonal holes. In addition, unlike in scRNA-seq where a single cell suspension needs to be prepared, SPBC requires an optimal cutting temperature (OCT) compound embedded or paraffin-embedded sample of tissue, which does not require high cell activity. Since the total amount of RNA expression and the efficiency of RNA permeabilization vary in different tumor tissues, 10X Genomics has introduced test slides to select the optimal permeabilization time for each sample.

To reduce the size of each spot to obtain a higher resolution, Slide-seq [31] and High-definition spatial transcriptomics (HDST) [32] were born. In Slide-seq, SOLiD sequencing chemistry [33] which requires the use of a bespoke fluidics-coupled microscope is applied for decoding beads, then the tissue is placed on a thin layer of beads for RNA capture with 10 μm resolution. The newest Slide-seqV2 [34] mixed advances in library production, bead manufacturing, and array indexing to achieve RNA capture efficiency ~ 50% that of single-cell RNA-seq data. As for HDST, an Illumina bead array is used to enable the SPBC to achieve a resolution of 2 μm [32]. Such beads are already much smaller than ordinary tumor cells, providing the possibility of subcellular resolution. Such high resolution allows this technology to greatly advance the application of spatial solved transcriptomics Meanwhile, some beads are strewn across cells, resulting in a reduced amount of captured RNA and reducing the infection of housekeeping RNA for cell-to-cell contacts analysis.

Different from SPBC, DNA barcodes will either be supplied to or recycled from specific tissue regions in another class of methods, termed selective barcoding (SB). GeoMx Digital Spatial Profiling (DSP) [35] and ZipSeq [36] were developed on this basis. DSP was also used for spatial solved proteomic analysis and ZipSeq expanded transcriptomics to the temporal dimension. Regrettably, they were not applied on a large scale due to the high cost.

This technology has enabled a leap in resolution accuracy, helping to understand the mode of action of tumor cells at the subcellular level. More and more commercial reagents and instruments are gradually penetrating the laboratory. In our opinion, three challenges remain with this technology. First, after enzymatic digestion, RNA moves laterally in tissues, causing misaligned capture and interfering with the precision of RNA spatial information. Tissue pretreatment like Hydrogel-Tissue is needed to reduce this movement. Second, large-scale hybrid reverse transcription may lead to distortion of the original gene expression information. Third, the high resolution may make the differences in RNA expression captured between each bead small in certain regions of high RNA expression, leading to redundancy in the data, special data processing tools are needed to help solve this problem.

## Advanced algorithms to process and analysis the dataset

When applied to spatially solved transcriptomics data, traditional analytical methods like Gene-category enrichment analysis (GCEA) and Gene Ontology (GO) might create a considerable false-positive bias [37]. In addition, Increased data volume leads to significantly longer analysis times. Consequently, novel algorithms are developed. For ISS methods, He et al. [38] introduced an unsupervised and annotation-free framework, termed ClusterMap, which defined the task as a point pattern, and found significant biological structures by density peak clustering (DPC). Cable et al. [39] developed robust cell type decomposition (RCTD), which could spatially map cell types and thus defined the spatial components of cell identity in Slide-seq and Visium datasets of the mouse brain. SPARK [40] directly models spatial count data through generalized linear spatial models and uses a computationally efficient algorithm based on penalized quasi-likelihood to enable the measurement of tens of thousands of genes. What’s more exciting, SPARK is 10 times more efficient than the traditional method when analyzing 4 published datasets of spatially solved transcriptomics. The advent of these aforementioned algorithms has helped to reveal more biological principles that will be applied to tumor research in the future as well.

## The emergence of web databases and open source tools

The diversity and complexity of strategies for data analysis, as well as the higher cost of being a new technology, have limited the exploitation of spatially solved transcriptomics datasets by researchers. The emergence of some relevant web databases is a good solution to this problem. Fan et al. [41] introduced SpatialDBC (https://www.spatialomics.org/SpatialDB), a database with 5 species, and 24 datasets, generated by eight spatially resolved transcriptomic techniques. SpatialDB is also a user-friendly online application that allows researchers to visualize and compare spatially resolved transcriptomic data. RNAlocation v2.0 (http://www.rnalocate.org/) has many updates to the original version that allows for the addition and reorganization of RNA information involving RNA subcellular localization conditions and descriptive figures for method, RNA homology information, and RNA interaction [42]. Existing databases are more focused on biological research, and we expect the emergence of cancer research-related databases like GEO and TCGA in the future. In addition, the development and application of open source tools such as STUtility [43], ST Viewer [44], and so on, make data processing and visual analysis easier.

## SRT technology is used in different tumor and other disease studies

As SRT technology continues to mature, it has been applied to the study of many types of solid tumors, including breast cancer, prostate cancer, melanoma, and liver cancer. Researchers have done a lot of exciting work with SRT technology.

**Fig. 2:**
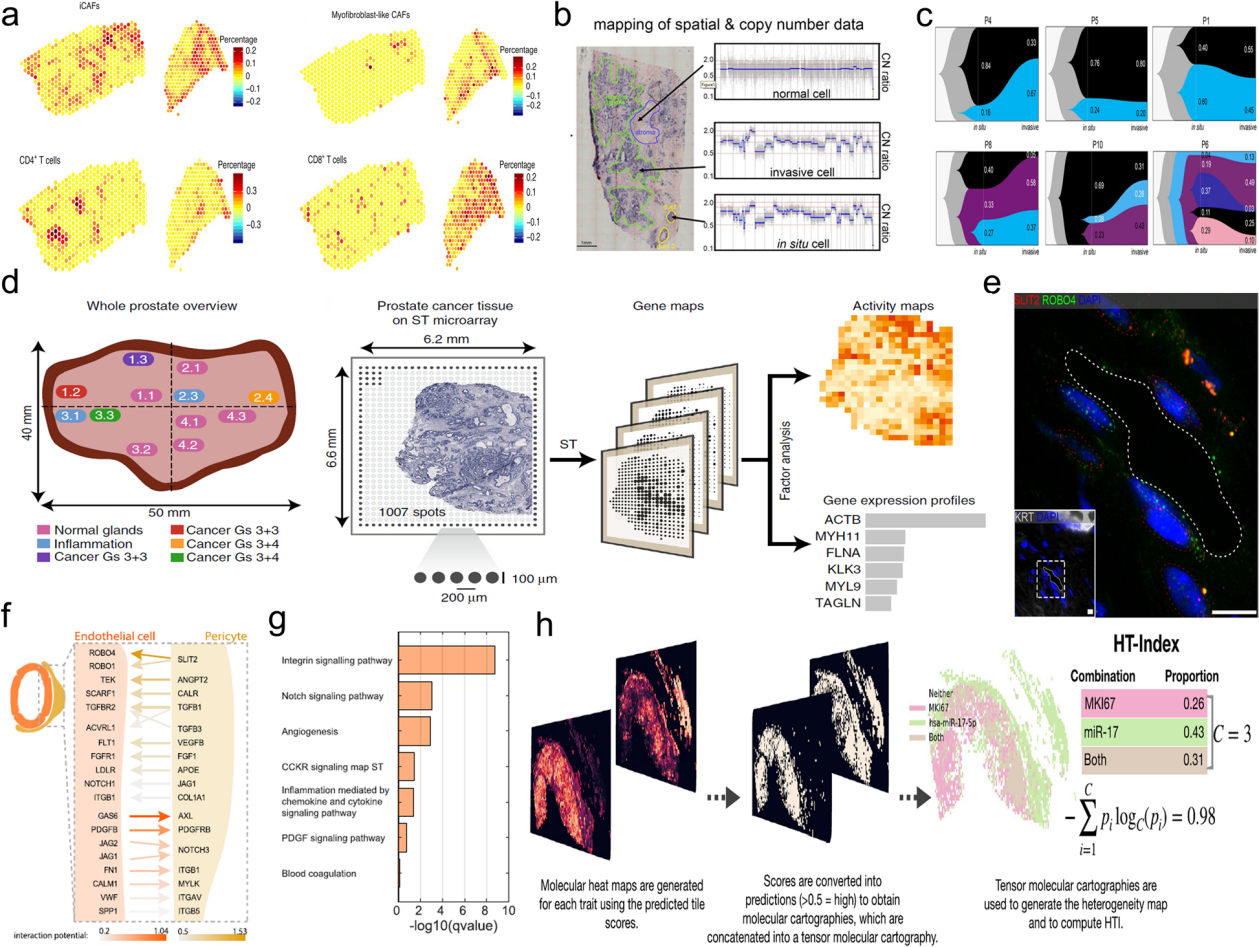
**a** In the research of Wu et al. (2021), a Pearson correlation analysis of the spatial distribution of different cell types within breast cancer tissues was performed. MyCAFs and iCAFs are negatively correlated in spatial distribution, and iCAFs with appear to co-localize spatially with T cells. **b** In Casasent et al. (2018) research, breast tumor cells were divided into normal, invasive, and in situ cell on pathological images. **c** Casasent et al. (2018) used Timescape to plot clonal lineages of the major tumor subpopulation, with common ancestors indicated in grey and clonal frequencies labeled for the in situ and invasive regions. The results demonstrate that genome evolution occurs within the ducts before the tumor cells escape the basement membrane. **d** Berglund et al. (2018) selected several regions with different pathological annotations b. Berglund et al. (2018) sequenced the spatial transcriptomics of different tissues separately and selected 10 RNAs by factor analysis, with each factor corresponding to an activity map. **e** Massalha et al. (2020) found the expression of the ligand SLIT2 mRNAs (red dots) in pericytes (marked by red dashed lines) and ROBO4 mRNAs (green dots) in endothelial cells (marked by green dashed lines). **f** Massalha et al. (2020) used the NicheNet to detect the interaction between pericytes and endothelial cells. **g** Massalha et al. (2020) performed an enrichment analysis of the highly correlated pathways and replicated it in multiple samples from different patients. **h** In Levy-Jurgenson et al. (2020) study, Generate tensor molecular thermograms from pathological sections, subsequently generate inhomogeneous distribution maps of cells, and generate heterogeneity indices using the formula

In breast cancer research, researchers identified moderate negative Pearson correlations between cancer-associated myofibroblasts (myCAFs) and cancer-associated inflammatory fibroblasts (iCAFs). MyCAFs were enriched in infiltrative cancer areas while iCAFs appeared to co-localize with several lymphocyte populations (Fig. 2a). In further research, receptor-ligand apical analysis of these cell co-localization regions revealed enrichment of immunoregulatory iCAF ligands and cognate T cell receptors nearby, including chemokines, transforming growth factor-β, the complement pathway, and lymphocyte inhibitory/activation molecules (LTB-LTBR, TNFSF14-LTBR, and LTB-CD40, VTCN1/B7H4-BTLA). They also found that PD-L1/PD-1 and PD-L2/PD-1 were coexpressed in spots enriched for LAM2 cells and CD4+/CD8+ T cells across multiple types of breast cancer, demonstrating that these cells are likely to have a role in immunoregulation [5]. In another study on gene copy variation in the progression of ductal carcinoma in situ to invasive ductal carcinoma of the breast, Casasent et al. [45] challenged the traditional clonal lineage model of gene mutations occurring during the invasion and the evolutionary bottleneck model by preserving the spatial context of individual tumor gene copies in tissue sections (Fig. 2c), and their study established a new model of polyclonal infiltration of tumors, and of course, the same idea can be applied to transcriptome studies of invasive tumors. In a spatial transcriptomics study of HER2-positive breast cancer [46], the researchers correlated pathologists with RNA expression-based clusters and found a high degree of consistency, and likewise found that data-driven expression-based clustering captured signals that were missed by visual inspection. They have also used spatial transcriptomic data to automatically generate pathological annotations of HER2-positive breast cancer, the same approach was also applied to the annotation of invasive ductal carcinoma pathology [47].

In digestive system malignancy research, Massalha and his colleagues used SRT technology to demonstrate that the interaction between endothelial cells and their attached pericytes plays an important role in the internal vascularization of malignant tumors of the liver [48]. In another study on pancreatic tumors, Moncada et al. [49] identified spatially restricted enrichment of ductal cells, macrophages, dendritic cells, and cancer cell subpopulations by multimodal intersection analysis (MIA) and demonstrated co-localization of inflammatory fibroblasts and cancer cells expressing stress response gene modules. Notably, they also detected a subpopulation of tumors with specific antigen presentation or apol1 hyper/hypoxia gene signatures not identified in previous single-cell sequencing studies and confirmed the existence of the subpopulation by dual fluorescein assays. In a recent study, scientists have proposed a genome atlas combining single-cell CITE-seq, spatially resolved transcriptomics, and spatially resolved proteomics. By integrating data sets from different histologies, the identification and localization of all hepatocytes are made more reliable. They also revealed the respective spatially resolved cellular niches of these macrophages and the microenvironmental circuitry driving their unique transcriptomic properties [50]. Although this innovative research method is currently only used to describe the structure of normal liver tissue, it will also become a trend in cancer research in later years.

In melanoma research, Toki et al. [51] used the DSP platform to classify the melanoma lymph node tissue section into CD68 + compartment, CD45+ compartment, and tumor compartment. Further studies showed that high CD8+ T cell counts in the CD65+ compartment were associated with prolonged OS and PFS, while this association was not found in the CD45+ compartment. To discover more prognosis-related molecules, the authors selected multiple cut-off points within different cells. They found several predictors of longer PFS such as CD8, CD3, TIM3, HLADR, CD11c, B2M, and PDL1. In another research, Kim et al. [52] applied SRT technology to gene expression profiling in melanoma lymph node metastases. They used tSNE to visualize the expression profiles after factor analysis, generated factor activity maps, and identified RNAs that were highly expressed in different strains of melanoma. In addition, they noted that PMEL and SPP1 were overexpressed in the tumor cell cluster whereas lymphoid tissue regions far away from and near, the tumor cell areas were characterized by expression of the immune-related genes CD74 and IGLL5, respectively. Their work has led to a deeper understanding of the intratumoral heterogeneity of melanoma.

In addition to tumor research, SRT technology was being used more often in the exploration of neurological diseases. Chen et al. [53] used the human APP knock-in APP^NL-G-F^ mouse model to probe transcriptomic changes during the progression of Alzheimer’s disease. They discovered a multicellular co-expressed gene network of 57 Plaque-Induced Genes (PIGs) that define a series of coordinated and tentatively defined the role of various types of cells contained within the network in the process of amyloid plaque formation and the interaction pattern between them. Gregory et al. [54] identified postmortem cerebral cortical tissue from a patient with myeloproliferative sclerosis using the SRT technique and found a spatial transcriptional imbalance between GRM3 and USP47. Although the resolution of 100 μm and the high degree of autolysis of tissue RNA after death had a major impact on the accuracy of the results, this study pioneered the application of SRT technology to human brain tissue. In a study of the glioma tumor microenvironment, Vidhya et al. [55] analyzed transcriptomic data to identify a subpopulation of HMOX1+ myeloid cells that release interleukin-10, which mediates T-cell depletion, leads to immunosuppression, and affects tumor progression.

## Some ideas on the application of SRT to oncology research

ScRNA-seq has played an important role in tumor research, including probing tumor heterogeneity [56], predicting tumor progression [57], and identifying specific subgroups [58]. However, scRNA-seq tissue processing methods, both the traditional FACS technology [59] and the more advanced nanogrid technology [60], have made the lack of spatial information of gene expression due to homogenization. Today SRT can preserve RNA positional information at a higher resolution, facilitating the understanding of tumor cell type and functional differences in different regions and further discovering heterogeneity within and between tumors.

The various methods of SRT mentioned above have been used to varying degrees in cancer research. SPBC is useful for developing large-scale tumor SRT databases because it integrates sequencing and pathology sectioning with high data throughput [61]. Due to the subcellular-level resolution, HDST is appropriate for high-precision expression profiling of individual tumor cells [46]. The image-based methods, researchers are using more often for complex neurodevelopment because of the advantages of visualization [11], and we believe that these methods have unique strength for the study of neurological tumors as well.

Since the application of SRT to oncology research is still in the early stage of exploration and there are not yet some fixed research methods, we propose several main application directions based on the understanding of previous studies to provide ideas for investigators.

### Further search for cell subgroups after unsupervised clustering

Differential gene expression analysis of tumor and paraneoplastic tissues has become an important tool in tumor bioinformatics research to help find coding or non-coding RNAs that play an important role in tumor development. In transcriptomics at the level of gene expression, SRT allows the working mechanism and working regions of coding or non-coding RNAs to be more tangible through the preservation of gene expression location information.

In transcriptomics studies at the cellular level, some large research projects have distinguished different types of tumor-associated cells and found their specific biomarkers after large-scale single-cell sequencing of all cells in regional cancer tissues and downscaling and clustering analysis of sequencing data using algorithms such as t-SNE or UMAP [62], which is undoubtedly a great breakthrough for further delineation of molecular typing of different kinds of tumors and provides ideas for individualized targeted therapy for tumors. However, the workload and cost of sequencing thousands or even tens of thousands of single cells are huge, and the differences in the pre-sequencing pretreatment received by different cells can also lead to questions about the authenticity of the sequencing results. Although SRT technology is still not a complete replacement for such research methods, it is slowly entering the minds of researchers due to its simplified workflow.

In the mapped spatial transcriptional profiles of tumor cells, the enrichment of some RNAs makes it easy to identify regions with active molecular pathways and has led to a clearer direction for studying the role of these aberrantly expressed RNA molecules in tumors. Berglund et al. [61] in their study analyzed a tissue section containing a tumor and performed a factor analysis of its spatial transcriptomics sequencing results. They identified a total of 10 mRNAs including KLK3, KLK2, MSMB, and ACPP as classification factors (Fig. 2d), and performed hierarchical clustering of these factors into four major categories corresponding to cancer, PIN, inflammatory, and normal gland tissues in the annotated pathology. This approach became the basis for automated tissue molecular annotation. Different annotated tissues have unique gene expression statuses, for instance, there was an enrichment of SPINK1 and PGC in cancer tissues, as well as deletion of ACPP. They then applied this type of molecular annotation to other tissue sections and found that the annotation results were similar to pathological annotation. They also found differences in the tumor center and peripheral expression. TAGLN, HLA, and ACTB are highly expressed in the peri-cancerous area which may be closely associated with tumor metastasis. Along the same lines, pancreatic tumor tissue was divided into cancer region, pancreatic tissue, duct epithelium, stroma [49], and melanoma lymph node tissue was classified into CD68 + compartment, CD45+ compartment, and tumor compartment [51]. Researchers have further built on this foundation by searching for specific molecular phenotypes and combining them with unknown patterns of cellular interaction or clinical prognosis to achieve a classification of tumor cell subpopulations. For example, Sinjab [63] et al.demonstrated that transcriptomic features span normal tissue regions to reach lung adenocarcinoma (LUAD), where elevated expression of CD24 in epithelial cells drives primary tumor features. Their study helps to identify cell populations, states, and phenotypes in the geographical and ecological progression of LUAD from the lung, including high-potential targets for early interception.

A relatively fixed pattern exists for the analysis of spatial gene expression differences in SRT data. Firstly, spatial enrichment of some genes is found using algorithms such as SVG, and common tools include SpatialDE [64] and Trendsceek [65]. Then different functional regions are sorted out according to the expression of specific genes, corresponding to abnormal metabolism, hypoxia response, vascular neogenesis, etc. SpaGCN [66] helps to realize this step. Finally, the clustered cells were mapped to different functional areas and some cell subpopulations with specific functions and their biomarkers were found.

Perhaps selecting multiple regions of tumor tissue for traditional single-cell sequencing would yield the same findings, but spatial transcriptomics seems to be more advantageous for interpreting tissue in junctional regions, especially when tumor boundaries are unclear.

However, unlike traditional single-cell sequencing, SRT appears to utilize a relatively fixed size point, rather than cells in the original sense, but even cell sizes and mRNA expression are different for the same type of cells. When spatial transcriptome resolution is not improved to the single-cell level, using SRT technology instead of traditional single-cell sequencing is likely to result in an overlap of cellular expression within a single point. Therefore, it becomes important to deal with the precise relationship between points and individual cells. Cell2location [67] and tangram [68] could apply deconvolution analysis to determine the type of cells contained in each point. Moreover, due to the limitations of sequencing technology, some low-expressed genes may not be detected and tangram [68] helps to predict the expression of these genes in various types of cells. All of the above techniques make the analysis of SRT data more realistic and convincing.

There is no quantitative index to visualize tumor heterogeneity in the aforementioned studies, and interestingly, Levy-Jurgenson et al. [54] proposed a new concept called heterogeneity index (HTI). They classified tumor cells into three categories: high expression of MKI67, high expression of miR-17, and high expression of MKI67 and miR-17. The uniform spatial distribution of these three categories of tumor cells corresponds to a higher HTI (Fig. 2 h). HTI can quantify the level of heterogeneity of a given image from a tensor molecular map. They also demonstrated a high correlation between HIT and breast cancer prognosis, a finding provided.

The analysis of spatial differences in RNA expression in tumor tissues is not just an improvement on scRNA-seq, but a major step forward in the search for important molecules in the mode of action of tumor cells. We expect that more HIT calculation methods for other types of tumors will be proposed by researchers, advancing SRT in the field of oncology research and achieving fundamental breakthroughs.

### Exploring heterotypic cell interactions

Tumor heterogeneity is largely reflected in heterotypic cell interactions in the tumor microenvironment, and previous studies have found that Cancer-associated fibroblasts (CAFS) are associated with poor prognosis [69, 70] while tumor-infiltrating lymphocytes (TILs) aggregation predicts high response to neoadjuvant chemotherapy [71]. SRT predicts the mode of action of heterotypic cells in the tumor microenvironment by monitoring the spatial expression of different cellular receptors and ligands and the regional enrichment of heterotypic cellular markers.

In a recent study, Massalha et al. [48] identified endothelial cell-peripheral cell interactions in liver malignancies. They first identified the co-localization of endothelial cells with pericytes on a spatial map. They then applied NicheNet [67], a computer technique that predicts ligand-receptor interactions based on downstream target gene activation, to demonstrate that the pericyte SLIT2 ligand and the endothelial ROBO receptor form the SLIT-ROBO signaling pathway and, together with other pathways, promote angiogenesis within malignant tumors (Fig. 2e-g). Most likely, this pathway will be a new site for targeted therapy in liver tumors. In breast cancer studies [5], spatial distribution correlation between fibroblasts and lymphocytes has also been found, and enrichment of multiple receptor-ligand pathways was identified. In addition, researchers have discovered evidence of diverse cellular interactions in the tumor microenvironment in other types of cancers, such as malignant melanoma [72] and prostate cancer [61].

For the study of heterotypic cell interaction mechanisms within different types of tumors, the application of SRT technology may vary depending on the tissue morphology and cell receptor expression, but a general idea exists. First, by investigating the spatial correlation of the distribution of heterotypic cells in the region, the cell-cell proximity network or fraction is used to identify the two cell types that are more likely to be associated (positively or negatively) in the course of tumor biology, thus allowing for a narrower and more targeted study. Then some biological processes that may exist between selected cells are identified through previous studies or signaling pathways that may intersect between cells are predicted using bioinformatics methods. Finally, to accurately verify the reliability of the pathway presence, apical analysis of receptor-ligand expression was performed using tools such as cellphoneDB [73] to better understand the mode of action between heterotypic cells.

In our view, SRT technology applied to the study of heterotypic cell interactions has several advantages over conventional methods. Firstly, regional aggregation and co-localization between different cell types can be easily detected by the analysis of spatial differences in the expression of specific markers of them. Notably, this is in comparison to the findings from pathological sections alone, which tend to be more precise and easier to detect the distribution of heterogeneous cells in low-grade tumors. Secondly, most previous studies in this area have simply analyzed the expression of cellular receptors and ligands and then predicted the potential mode of action between cells. However, owing to the retention of spatial information, the signaling routes established between cellular receptor ligands are identified more intuitively, which reduces the false positive rate produced by the prediction calculation approach. Thirdly, SRT methods are also expected to assist in the investigation of three or more cell contact patterns, which will undoubtedly be a quantum leap in the field of oncology research.

### Fusion of expression profiles with pathology images

It is well known that pathology is the gold standard for the diagnosis of solid tumors. However, the emergence of SRT technology seems to shake the status of traditional pathology diagnosis. When drawing the spatial maps of prostate cancer transcriptomes, Berglund et al. [61] found that the cancer expression region exceeded the pathological annotation region marked by experienced pathologists. These “high-risk areas” over the portion may determine the scope of the surgery. The investigators also used the spatial transcriptome to automate the pathological annotation of HER-2-positive breast cancer [46] as well as invasive ductal carcinoma [47]. Although this method cannot replace pathological sections in the short term due to cost and may provide pathologists with some clinical decisions hereafter.

With the development of computer technology, deep learning has also been applied to oncology research. Bryan et al. [74] presented ST-Net, a deep learning method that captures high-resolution gene expression heterogeneity by combining spatial transcriptomics and histological pictures. ST-NET uses easily available hematoxylin eosin-stained histopathology images to predict spatial differences in the expression of 102 genes with a resolution of 100 μm, including GNAS, FASN, DDX5, XBP1, and other known breast cancer biomarkers. To reduce experimental noise, they averaged the gene expression values of each point with those of the adjacent points. ST-NET was directly applied to the interpretation of breast cancer HE images from the TCGA tumor database, the results demonstrate that this algorithm can be extended to the construction of breast cancer gene expression datasets without repeated training, and It is also applicable to the tumor genome, The same approach has also been well applied in the field of colon cancer research [75]. The widespread application of this technique requires a great deal of preliminary work, including the mapping of large-scale spatial transcriptions, the standardization of a large number of pathological sections, and the iterative training and continuous improvement of the algorithm. Also, it is worth thinking about how to reduce the impact of staining and decolorization on the tissue transcriptome. In the future, these methods will probably be widely employed in transcriptomics research and early diagnosis of cancer.

### A “pseudo-timeline” on the same tissue section to explore tumor progression

While SRT technology is maturing, time resolved transcriptomics is also entering the vision of researchers, and this approach is now more frequently used for transcriptomic alterations during embryonic development. In tumor research, since the spatial structure of the same tissue changes greatly over time, which makes it extremely difficult to compare transcriptome differences at different time points, most of the spatial transcriptomics studies at this stage stay at the cellular level. Researchers have also come up with some solutions to address this difficulty. One is constructing animal models with stable tumor size and intratumor structure to minimize transcriptome differences due to spatial structure. The other is selecting cells at different stages in the same tissue section for transcriptomic or genomic analysis, forming a “pseudo-time” axial approach to research. The former procedure seems to be more complicated and ensuring the survival of the model animals after sampling is a big challenge. Of course, the latter also has some drawbacks, as the transcriptome changes during tumor development cannot be fully reflected in a single tissue section at a certain stage.

Interestingly, researchers made a related attempt a few years ago. In studying the progression of ductal carcinoma in situ (DCIs) to invasive ductal carcinoma (IDC) in the breast, Casasent et al. [45] classified tumor cells into DCIs and IDC by observing whether they crossed the basement membrane on pathological images (Fig. 2b), then they used the spatial context of preserving gene copies of individual tumors in tissue sections to discover a direct genomic genealogy between in situ and invasive tumor subgroups and further demonstrate that the vast majority of mutations occur in ducts that do not become infiltrated (Fig. 2c). This research idea can be extended to other aspects of tumor research. For example, by observing changes in the number of relevant receptor expressions on the cell surface, the mechanisms of drug resistance to targeted drugs can be studied in depth. Another example is to predict multiple biological alterations occurring during metastasis by observing the transcriptome differences between primary and metastatic.

## Conclusion and prospects

Among lots of SRT methods, the SPBC technique has been the first to be commercialized and widely used in various studies due to its relatively simple method, mastered workflow, and low cost. Although HDST can achieve subcellular resolution, the need for special Illumina beads limits its applicability. The image-based method is also maturing, and it is expected to give full play to its visualization advantages and become the choice of a non-professional. In situ sequencing methods such as FISSEQ and STARmap can also achieve subcellular resolution and detect more genes, but are still limited to a few independent laboratories due to customized technology and lower experimental throughput. The continuous improvement of various technologies has also gradually overcome the two main difficulties of data throughput and resolution, and the emergence of open-source tools and network databases has greatly lowered the threshold of SRT research.

Despite the achievements of SRT technology in cancer research, there is still much room for future improvement as a novel research tool. Through SRT technology, the heterogeneity of each type of tumor can be reflected as a more quantitative index and correlated with tumor survival or treatment response, which facilitates tumor-related clinical research. The integration of SRT technology with computer technology is expected to lead to more automated and accurate staging of tumor tissues, reducing the work pressure and possible visual errors of pathologists. At the present stage, spatial transcriptomics research is more focused on the description of the original expression profile of tumors, and this part of the research results does not play many roles in clinical tumor treatment. The direction of future research can be expanded to tumor-targeted therapy and chemotherapy drug resistance mechanisms and SRT technology can indirectly benefit patients.

Some researchers have integrated SRT technology with spatial genomics and spatial proteomics through mass spectrometry and multi-bit immunofluorescence to gain more insight into cellular metabolic processes and protein post-translational modification mechanisms [76–79]. However, such multi-omics fusion approaches have not been widely carried out due to the complexity of the research methods and technical bottlenecks. We come up with a bold idea, in SPBC methods, improving hybridization probes to combine more biomolecules including DNA coding strands, non-coding RNAs, and proteins, enabling spatial multi-omics studies of the same tissue. The proposal of DBiT-seq [80] also provides a new strategy for multi-omics fusion studies of tumor tissues. As the spatial multi-omics research system continues to mature, it will be applied to the precise localization of tumor mutated genes, the detection of tumor cell communication networks, and the retrieval of abnormal proteins in the future, helping researchers deepen their understanding of cancer pathogenesis and progression mechanisms, and driving the discovery of new therapeutic targets and more advanced treatments.

## Data Availability

All available data and material can be accessed.

## Abbreviations

SRT: Spatially resolved transcriptomics
scRNA-seq: Single-cell RNA sequencing
FISH: Fluorescence in situ hybridization
MERFISH: Multiplexed error-robust FISH
ISS: In situ Sequencing
RCA: Rolling circle amplification
SPBC: Solid phase-based capture
HDST: High-definition spatial transcriptomics
DSP: Digital Spatial Profiling
RTD: Robust cell type decomposition
myCAFs: Cancer-associated myofibroblasts
iCAFs: Cancer-associated inflammatory fibroblasts
MIA: Multimodal intersection analysis
LUAD: Lung adenocarcinoma
DCIs: Ductal carcinoma in situ
IDC: Invasive ductal carcinoma
OCT: Optimal cutting temperature

## References

[1] Puram SV, Tirosh I, Parikh AS, Patel AP, Yizhak K, Gillespie S (2017). Single-cell transcriptomic analysis of primary and metastatic tumor ecosystems in head and neck Cancer. Cell.

[2] Lambrechts D, Wauters E, Boeckx B, Aibar S, Nittner D, Burton O (2018). Phenotype molding of stromal cells in the lung tumor microenvironment. Nat Med.

[3] van Galen P, Hovestadt V, Wadsworth Ii MH, Hughes TK, Griffin GK, Battaglia S (2019). Single-cell RNA-Seq reveals AML hierarchies relevant to disease progression and immunity. Cell.

[4] Wagner J, Rapsomaniki MA, Chevrier S, Anzeneder T, Langwieder C, Dykgers A (2019). A single-cell atlas of the tumor and immune ecosystem of human breast Cancer. Cell.

[5] Wu SZ, Al-Eryani G, Roden DL, Junankar S, Harvey K, Andersson A (2021). A single-cell and spatially resolved atlas of human breast cancers. Nat Genet.

[6] Young MD, Mitchell TJ, Vieira Braga FA, Tran MGB, Stewart BJ, Ferdinand JR (2018). Single-cell transcriptomes from human kidneys reveal the cellular identity of renal tumors. Science (New York, NY).

[7] Kashyap A, Autebert J, Delamarche E, Kaigala GV (2016). Selective local lysis and sampling of live cells for nucleic acid analysis using a microfluidic probe. Sci Rep.

[8] Peng G, Suo S, Cui G, Yu F, Wang R, Chen J (2019). Molecular architecture of lineage allocation and tissue organization in early mouse embryo. Nature.

[9] Dekoninck S, Hannezo E, Sifrim A, Miroshnikova YA, Aragona M, Malfait M (2020). Defining the design principles of skin epidermis postnatal growth. Cell.

[10] Chen X, Sun YC, Zhan H, Kebschull JM, Fischer S, Matho K (2019). High-throughput mapping of Long-range neuronal projection using in situ sequencing. Cell.

[11] Zhang M, Eichhorn SW, Zingg B, Yao Z, Cotter K, Zeng H (2021). Spatially resolved cell atlas of the mouse primary motor cortex by MERFISH. Nature.

[12] Wang X, Allen WE, Wright MA, Sylwestrak EL, Samusik N, Vesuna S, et al. Three-dimensional intact-tissue sequencing of single-cell transcriptional states. Science (New York, NY). 2018;361(6400). 10.1126/science.aat5691. 10.1126/science.aat5691 PMC6339868 29930089

[13] Zhuang X (2021). Spatially resolved single-cell genomics and transcriptomics by imaging. Nat Methods.

[14] Larsson L, Frisén J, Lundeberg J (2021). Spatially resolved transcriptomics adds a new dimension to genomics. Nat Methods.

[15] Lubeck E, Coskun AF, Zhiyentayev T, Ahmad M, Cai L (2014). Single-cell in situ RNA profiling by sequential hybridization. Nat Methods.

[16] Chen KH, Boettiger AN, Moffitt JR, Wang S, Zhuang X (2015). RNA imaging. Spatially resolved, highly multiplexed RNA profiling in single cells. Science (New York, NY).

[17] Xia C, Fan J, Emanuel G, Hao J, Zhuang X (2019). Spatial transcriptome profiling by MERFISH reveals subcellular RNA compartmentalization and cell cycle-dependent gene expression. Proc Natl Acad Sci U S A.

[18] Wang G, Moffitt JR, Zhuang X (2018). Multiplexed imaging of high-density libraries of RNAs with MERFISH and expansion microscopy. Sci Rep.

[19] Chen F, Tillberg PW, Boyden ES (2015). Optical imaging. Expansion microscopy. Science (New York, NY).

[20] Moffitt JR, Hao J, Bambah-Mukku D, Lu T, Dulac C, Zhuang X (2016). High-performance multiplexed fluorescence in situ hybridization in culture and tissue with matrix imprinting and clearing. Proc Natl Acad Sci U S A.

[21] Eng CL, Lawson M, Zhu Q, Dries R, Koulena N, Takei Y (2019). Transcriptome-scale super-resolved imaging in tissues by RNA seqFISH. Nature.

[22] Kishi JY, Lapan SW, Beliveau BJ, West ER, Zhu A, Sasaki HM (2019). SABER amplifies FISH: enhanced multiplexed imaging of RNA and DNA in cells and tissues. Nat Methods.

[23] Codeluppi S, Borm LE, Zeisel A, La Manno G, van Lunteren JA, Svensson CI (2018). Spatial organization of the somatosensory cortex revealed by osmFISH. Nat Methods.

[24] Beliveau BJ, Joyce EF, Apostolopoulos N, Yilmaz F, Fonseka CY, McCole RB (2012). Versatile design and synthesis platform for visualizing genomes with Oligopaint FISH probes. Proc Natl Acad Sci U S A.

[25] Ke R, Mignardi M, Pacureanu A, Svedlund J, Botling J, Wählby C (2013). In situ sequencing for RNA analysis in preserved tissue and cells. Nat Methods.

[26] Lee JH, Daugharthy ER, Scheiman J, Kalhor R, Yang JL, Ferrante TC (2014). Highly multiplexed subcellular RNA sequencing in situ. Science (New York, NY).

[27] Lee JH, Daugharthy ER, Scheiman J, Kalhor R, Ferrante TC, Terry R (2015). Fluorescent in situ sequencing (FISSEQ) of RNA for gene expression profiling in intact cells and tissues. Nat Protoc.

[28] Alon S, Goodwin D, Sinha A, Wassie A, Chen F, Daugharthy E (2020). Expansion sequencing: spatially precise. Situ transcriptomics in intact biological systems.

[29] Ståhl PL, Salmén F, Vickovic S, Lundmark A, Navarro JF, Magnusson J (2016). Visualization and analysis of gene expression in tissue sections by spatial transcriptomics. Science (New York, NY).

[30] Salmén F, Ståhl PL, Mollbrink A, Navarro JF, Vickovic S, Frisén J (2018). Barcoded solid-phase RNA capture for spatial transcriptomics profiling in mammalian tissue sections. Nat Protoc.

[31] Rodriques SG, Stickels RR, Goeva A, Martin CA, Murray E, Vanderburg CR (2019). Slide-seq: a scalable technology for measuring genome-wide expression at high spatial resolution. Science (New York, NY).

[32] Vickovic S, Eraslan G, Salmén F, Klughammer J, Stenbeck L, Schapiro D (2019). High-definition spatial transcriptomics for in situ tissue profiling. Nat Methods.

[33] Gunderson KL, Kruglyak S, Graige MS, Garcia F, Kermani BG, Zhao C (2004). Decoding randomly ordered DNA arrays. Genome Res.

[34] Stickels RR, Murray E, Kumar P, Li J, Marshall JL, Di Bella DJ (2021). Highly sensitive spatial transcriptomics at near-cellular resolution with slide-seqV2. Nat Biotechnol.

[35] Merritt CR, Ong GT, Church SE, Barker K, Danaher P, Geiss G (2020). Multiplex digital spatial profiling of proteins and RNA in fixed tissue. Nat Biotechnol.

[36] Hu KH, Eichorst JP, McGinnis CS, Patterson DM, Chow ED, Kersten K (2020). ZipSeq: barcoding for real-time mapping of single cell transcriptomes. Nat Methods.

[37] Fulcher BD, Arnatkeviciute A, Fornito A (2021). Overcoming false-positive gene-category enrichment in the analysis of spatially resolved transcriptomic brain atlas data. Nat Commun.

[38] He Y, Tang X, Huang J, Ren J, Zhou H, Chen K (2021). ClusterMap for multi-scale clustering analysis of spatial gene expression. Nat Commun.

[39] Cable DM, Murray E, Zou LS, Goeva A, Macosko EZ, Chen F, et al. Robust decomposition of cell type mixtures in spatial transcriptomics. Nat Biotechnol. 2021. 10.1038/s41587-021-00830-w. 10.1038/s41587-021-00830-w PMC8606190 33603203

[40] Sun S, Zhu J, Zhou X (2020). Statistical analysis of spatial expression patterns for spatially resolved transcriptomic studies. Nat Methods.

[41] Fan Z, Chen R, Chen X (2020). SpatialDB: a database for spatially resolved transcriptomes. Nucleic Acids Res.

[42] Cui T, Dou Y, Tan P, Ni Z, Liu T, Wang D, et al. RNALocate v2.0: an updated resource for RNA subcellular localization with increased coverage and annotation. Nucleic Acids Res. 2021. 10.1093/nar/gkab825. 10.1093/nar/gkab825 PMC8728251 34551440

[43] Bergenstråhle J, Larsson L, Lundeberg J (2020). Seamless integration of image and molecular analysis for spatial transcriptomics workflows. BMC Genomics.

[44] Fernández Navarro J, Lundeberg J, Ståhl PL (2019). ST viewer: a tool for analysis and visualization of spatial transcriptomics datasets. Bioinformatics (Oxford, England).

[45] Casasent AK, Schalck A, Gao R, Sei E, Long A, Pangburn W (2018). Multiclonal invasion in breast tumors identified by topographic single cell sequencing. Cell.

[46] Andersson A, Larsson L, Stenbeck L, Salmén F, Ehinger A, Wu S (2020). Spatial deconvolution of HER2-positive breast tumors reveals novel intercellular relationships.

[47] Yoosuf N, Navarro JF, Salmén F, Ståhl PL, Daub CO (2020). Identification and transfer of spatial transcriptomics signatures for cancer diagnosis. Breast Cancer Res.

[48] Massalha H, Bahar Halpern K, Abu-Gazala S, Jana T, Massasa EE, Moor AE (2020). A single cell atlas of the human liver tumor microenvironment. Mol Syst Biol.

[49] Moncada R, Barkley D, Wagner F, Chiodin M, Devlin JC, Baron M (2020). Integrating microarray-based spatial transcriptomics and single-cell RNA-seq reveals tissue architecture in pancreatic ductal adenocarcinomas. Nat Biotechnol.

[50] Guilliams M, Bonnardel J, Haest B, Vanderborght B, Wagner C, Remmerie A (2022). Spatial proteogenomics reveals distinct and evolutionarily conserved hepatic macrophage niches. Cell.

[51] Toki MI, Merritt CR, Wong PF, Smithy JW, Kluger HM, Syrigos KN (2019). High-Plex predictive marker discovery for melanoma immunotherapy-treated patients using digital spatial profiling. Clin Cancer Res.

[52] Thrane K, Eriksson H, Maaskola J, Hansson J, Lundeberg J (2018). Spatially resolved transcriptomics enables dissection of genetic heterogeneity in stage III cutaneous malignant melanoma. Cancer Res.

[53] Chen WT, Lu A, Craessaerts K, Pavie B, Sala Frigerio C, Corthout N (2020). Spatial transcriptomics and in situ sequencing to study Alzheimer's disease. Cell.

[54] Gregory JM, McDade K, Livesey MR, Croy I, Marion de Proce S, Aitman T (2020). Spatial transcriptomics identifies spatially dysregulated expression of GRM3 and USP47 in amyotrophic lateral sclerosis. Neuropathol Appl Neurobiol.

[55] Ravi VM, Neidert N, Will P, Joseph K, Maier JP, Kückelhaus J (2022). T-cell dysfunction in the glioblastoma microenvironment is mediated by myeloid cells releasing interleukin-10. Nat Commun.

[56] Zong C, Lu S, Chapman AR, Xie XS (2012). Genome-wide detection of single-nucleotide and copy-number variations of a single human cell. Science (New York, NY).

[57] Navin N, Kendall J, Troge J, Andrews P, Rodgers L, McIndoo J (2011). Tumour evolution inferred by single-cell sequencing. Nature.

[58] Song Y, Tian T, Shi Y, Liu W, Zou Y, Khajvand T (2017). Enrichment and single-cell analysis of circulating tumor cells. Chem Sci.

[59] Baslan T, Kendall J, Rodgers L, Cox H, Riggs M, Stepansky A (2012). Genome-wide copy number analysis of single cells. Nat Protoc.

[60] Gao R, Kim C, Sei E, Foukakis T, Crosetto N, Chan LK (2017). Nanogrid single-nucleus RNA sequencing reveals phenotypic diversity in breast cancer. Nat Commun.

[61] Berglund E, Maaskola J, Schultz N, Friedrich S, Marklund M, Bergenstråhle J (2018). Spatial maps of prostate cancer transcriptomes reveal an unexplored landscape of heterogeneity. Nat Commun.

[62] Karthaus WR, Hofree M, Choi D, Linton EL, Turkekul M, Bejnood A (2020). Regenerative potential of prostate luminal cells revealed by single-cell analysis. Science (New York, NY).

[63] Sinjab A, Han G, Treekitkarnmongkol W, Hara K, Brennan PM, Dang M (2021). Resolving the spatial and cellular architecture of lung adenocarcinoma by multiregion single-cell sequencing. Cancer Discov.

[64] Svensson V, Teichmann SA, Stegle O (2018). SpatialDE: identification of spatially variable genes. Nat Methods.

[65] Edsgard D, Johnsson P, Sandberg R (2018). Identification of spatial expression trends in single-cell gene expression data. Nat Methods.

[66] Hu J, Li XJ, Coleman K, Schroeder A, Ma N, Irwin DJ (2021). SpaGCN: Integrating gene expression, spatial location and histology to identify spatial domains and spatially variable genes by graph convolutional network. Nat Methods.

[67] Kleshchevnikov V, Shmatko A, Dann E, Aivazidis A, King HW, Li T, et al. Cell2location maps fine-grained cell types in spatial transcriptomics. Nat Biotechnol. 2022. 10.1038/s41587-021-01139-4. 10.1038/s41587-021-01139-4 35027729

[68] Biancalani T, Scalia G, Buffoni L, Avasthi R, Lu Z, Sanger A (2021). Deep learning and alignment of spatially resolved single-cell transcriptomes with tangram. Nat Methods.

[69] Lakins MA, Ghorani E, Munir H, Martins CP, Shields JD (2018). Cancer-associated fibroblasts induce antigen-specific deletion of CD8 (+) T cells to protect tumour cells. Nat Commun.

[70] Elyada E, Bolisetty M, Laise P, Flynn WF, Courtois ET, Burkhart RA (2019). Cross-species single-cell analysis of pancreatic ductal adenocarcinoma reveals antigen-presenting Cancer-associated fibroblasts. Cancer Discov.

[71] Dushyanthen S, Beavis PA, Savas P, Teo ZL, Zhou C, Mansour M (2015). Relevance of tumor-infiltrating lymphocytes in breast cancer. BMC Med.

[72] Hunter MV, Moncada R, Weiss JM, Yanai I, White RM (2021). Spatially resolved transcriptomics reveals the architecture of the tumor-microenvironment interface. Nat Commun.

[73] Efremova M, Vento-Tormo M, Teichmann SA, Vento-Tormo R (2020). CellPhoneDB: inferring cell-cell communication from combined expression of multi-subunit ligand-receptor complexes. Nat Protoc.

[74] He B, Bergenstråhle L, Stenbeck L, Abid A, Andersson A, Borg Å (2020). Integrating spatial gene expression and breast tumour morphology via deep learning. Nat Biomed Eng.

[75] Sirinukunwattana K, Domingo E, Richman SD, Redmond KL, Blake A, Verrill C (2021). Image-based consensus molecular subtype (imCMS) classification of colorectal cancer using deep learning. Gut.

[76] Schürch CM, Bhate SS, Barlow GL, Phillips DJ, Noti L, Zlobec I (2020). Coordinated cellular neighborhoods orchestrate Antitumoral immunity at the colorectal Cancer invasive front. Cell.

[77] Keren L, Bosse M, Thompson S, Risom T, Vijayaragavan K, McCaffrey E (2019). MIBI-TOF: A multiplexed imaging platform relates cellular phenotypes and tissue structure. Sci Adv.

[78] Jackson HW, Fischer JR, Zanotelli VRT, Ali HR, Mechera R, Soysal SD (2020). The single-cell pathology landscape of breast cancer. Nature.

[79] Goltsev Y, Samusik N, Kennedy-Darling J, Bhate S, Hale M, Vazquez G (2018). Deep profiling of mouse splenic architecture with CODEX multiplexed imaging. Cell.

[80] Liu Y, Yang M, Deng Y, Su G, Enninful A, Guo CC (2020). High-spatial-resolution multi-omics sequencing via deterministic barcoding in tissue. Cell.

